# Giant adrenal ganglioneuroma in children: a case report

**DOI:** 10.1007/s12672-022-00573-3

**Published:** 2022-10-14

**Authors:** Mingqiu Hu, Zhizhong Tang, Yong Cai, Xiaolu Yuan

**Affiliations:** 1 Department of Urology, MaomingPeoplès Hospital, Maoming, 525000 China; 2 Department of Computed Tomography, MaomingPeoplès Hospital, Maoming, 525000 China; 3 Department of Pathology, MaomingPeoplès Hospital, Maoming, 525000 China

**Keywords:** Ganglioneuroma, Adrenal ganglioneuroma, Adrenal glands, Computed tomography, Ganglioneuroma

## Abstract

**Background:**

Ganglioneuromas (GNs) arise from the Schwann cells, ganglion cells, and neuronal tissues, and are extremely rare, slow-growing, benign tumors. GN has usually grown very large when it is diagnosed since no specific clinical symptoms or laboratory findings indicating GN are available, especially when it occurs in the retroperitoneal space. Total resection of the tumor is the recommended treatment.

**Case summary:**

We present the imaging and pathological findings of a giant adrenal GN in a child. A 7-year-old boy suffered from nausea and postprandial vomiting for 1 week with no precipitating factors. There was no family history of any disease, and the boy did not suffer from any disease in the past. Biochemical examination showed normal results. Physical examination showed an immobilized palpable mass in the left abdominal area. Abdominal computed tomography revealed a 13 cm × 10 cm solid mass in the retroperitoneal space. The mass showed slight and heterogeneous enhancement after injection of a contrasting agent. The mass was surgically resected locally to address the embedded abdominal vessels, and the histopathological and immunohistochemical diagnosis of the mass was GN. After the surgery, the symptoms of nausea and vomiting were relieved, and no complications occurred.

**Conclusion:**

GN should be considered when a child presents with a giant retroperitoneal hypodense mass and the mass presents uneven and delayed enhancement. Histopathology is the golden standard for the diagnosis of GN. Currently, surgical excision is the optimal treatment.

## Introduction

Ganglioneuroma (GN) is a rare, benign tumor. It usually occurs in the posterior mediastinum and retroperitoneum. GNs originating from the adrenal glands, neck, and paravertebral sympathetic plexus region are relatively rare. Adrenal ganglioneuroma (AGN) is a slow-growing neoplasm located in the adrenal glands and accounts for 20–30% of all GN cases [1]. A giant AGN is very rare in the clinic. The management of a giant AGN is generally challenging for urological surgeons, especially before the diagnosis. Surgical resection of the giant retroperitoneal tumor is usually extremely difficult, involves high risk, and is a complex procedure [1]. Here, we report a case to assess the feasibility of removing the giant AGN.

## Case description

A 7-year-old boy with nausea and vomiting for 1 week was presented to the gastroenterology department of our hospital. The boy was healthy and there was no past medical history or family history of any disease. During the physical examination, a left immobilized palpable solid abdominal mass of 13 cm × 10 cm was detected. Since the mass possibly originated from the left adrenal gland, adrenal endocrinological examinations were conducted. Biochemical tests revealed that the levels of cortisol were 1.36 mg/dl, 17.68 mg/dl, and 5.82 mg/dl at 0 h, 8 h, and 16 h, respectively (normal ranges: 0–1.54 mg/dl, 7.2–18.2 mg/dl, and 2.75–6.65 mg/dl, respectively), and that of fibrinogen was 1.9 g/l (normal range: 2–4 g/l). The level of aldosterone (45.8 pg/ml) was in the normal range (normal range: 23.5-106.6 pg/ml). Similarly, the levels of ACTH were in the normal range (11.3 pg/ml, 33.2 pg/ml, and 23.6 pg/ml at 0 h, 8 h, and 16 h, respectively; normal range < 46 pg/ml).

**Fig. 1:**
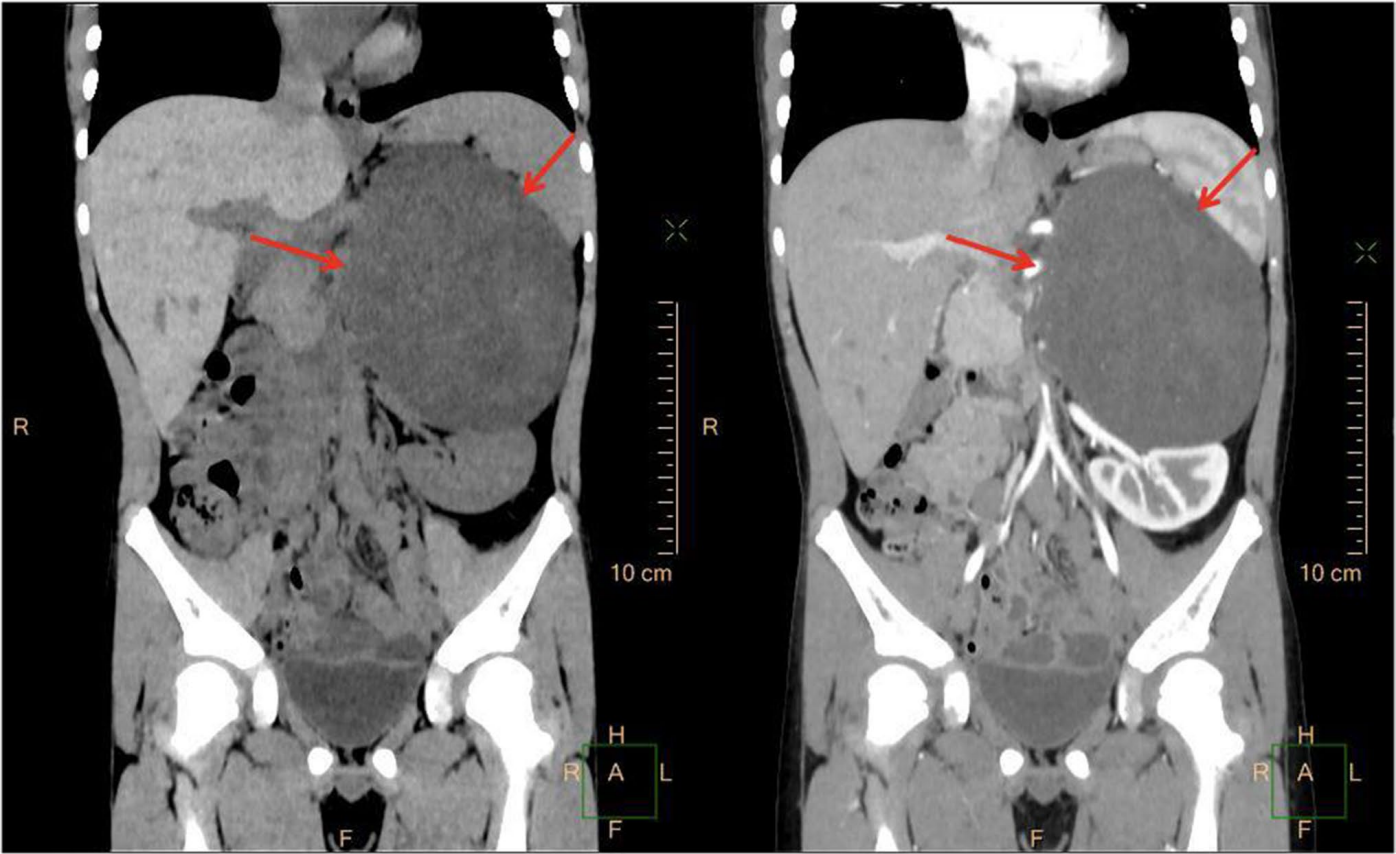
CT showed a large oval mass of homogenous soft-tissue density above the left kidney with slight enhancement

Abdominal computed tomography (CT) showed a large solid mass on the left retroperitoneum measuring approximately 10.1 cm × 9.3 cm × 13.4 cm in size (Fig. 1). The internal density of the mass was uneven, and the CT density was approximately 16–34 HU; The enhanced scan lesion showed an uneven, slight enhancement, and the CT densities of the arterial, portal, and delayed phases were approximately 16–39 HU, 17–60 HU, and 17–68 HU, respectively, and fewer nodules were observed in the lesion. A branch of a small blood vessel from the abdominal aorta supplied the tumor. The adjacent bowel and pancreas were compressed and displaced, and the left kidney was compressed down to the iliac crest level. The left adrenal gland was not visible, therefore, the possibility of a tumor originating from the left adrenal gland was high. The tumor was surgically removed. The pediatric patient was placed in the supine position, inclined 30 degrees to the right, and an oblique incision of about 25 cm in length was made under the left ribs. The lateral peritoneum was opened along the lateral side of the mesocolon, and the colon and colic mesentery were gently pushed medially to expose the tumor. During the surgery, the tumor was found to be located in the left retroperitoneum, the capsule was intact, and no adhesion to the diaphragm, posterior peritoneum, or upper pole of the kidney was noted. The mass was mobilized gently along with the capsule. The tumor received blood supply via a great vessel from the aorta; which was ligated and cut carefully. After removing the mass, the left retroperitoneal space was explored, and the left adrenal gland was not found.

**Fig. 2:**
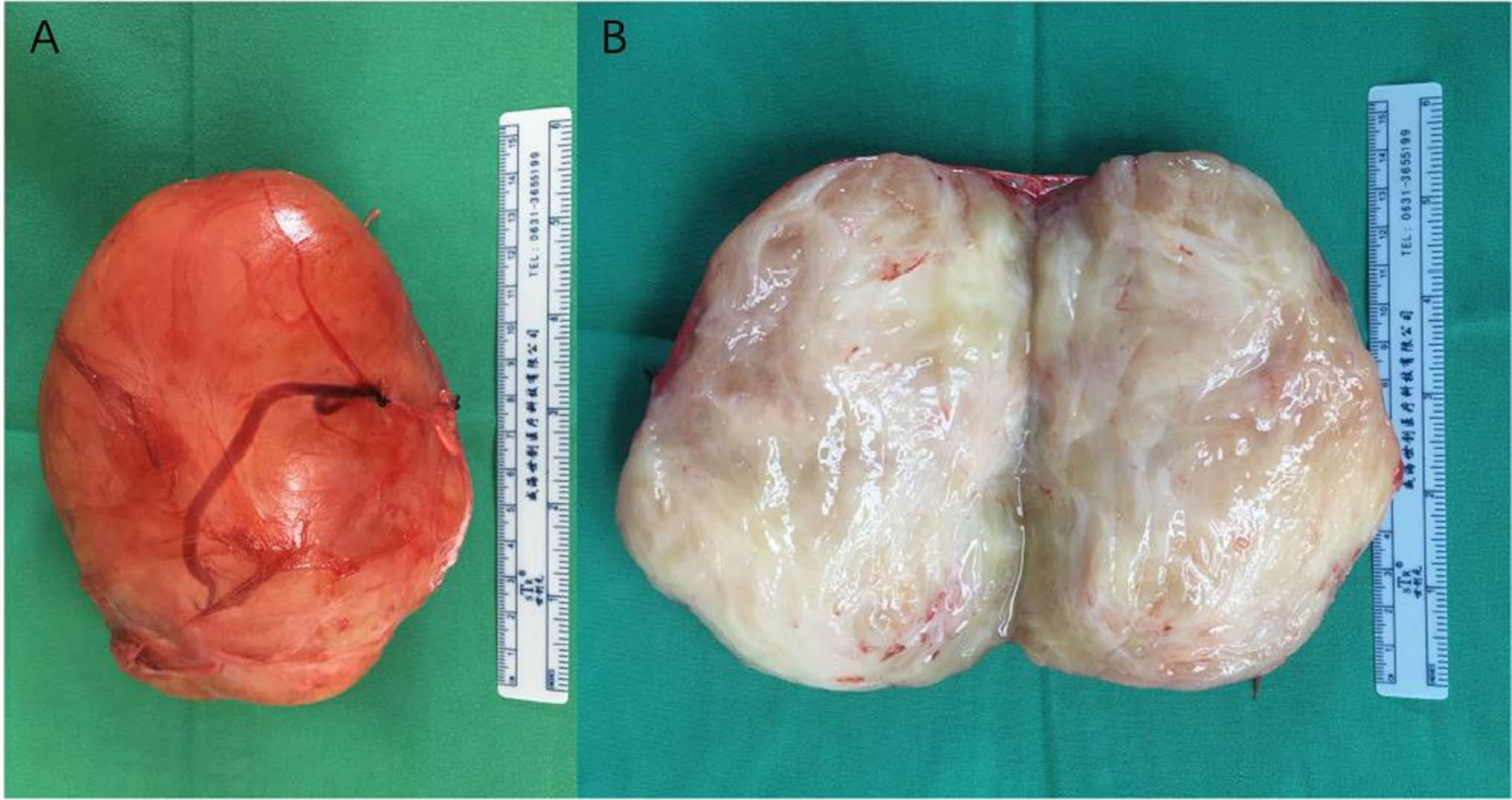
Excised specimen shows a giant adrenal ganglioneuroma which measured 13 cm × 8 cm × 8 cm. **A** Gross pathological appearance; **B** Cross-section appearance of the tumor

**Fig. 3:**
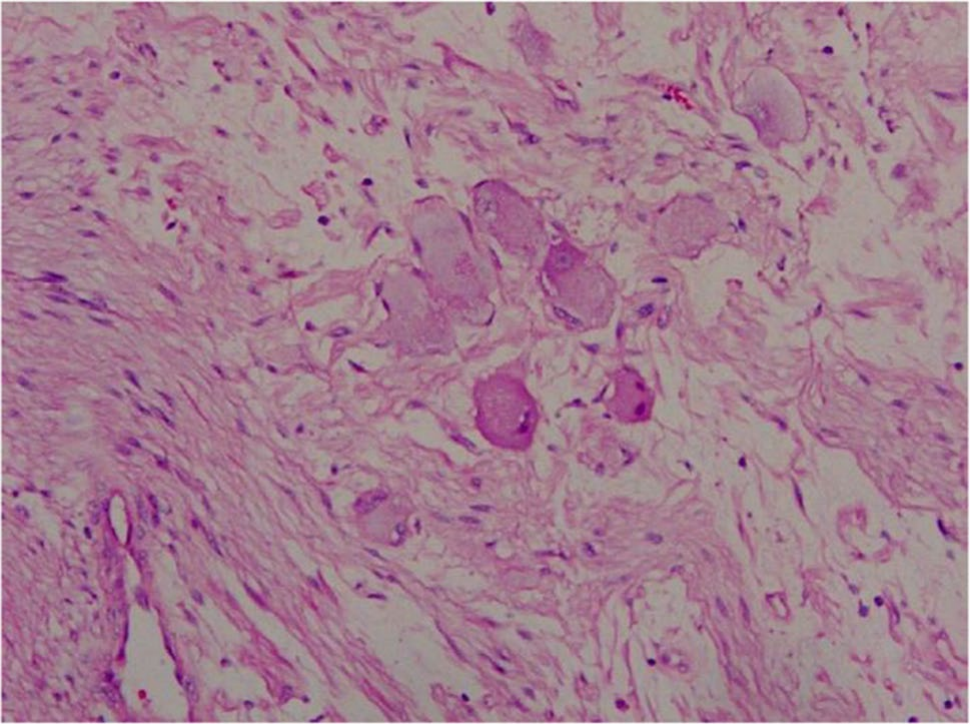
Histopathology of ganglioneuroma. Microscopically showed ganglion cells and Schwann cells (hematoxylin and eosin stain). Original magnification × 200

**Fig. 4:**
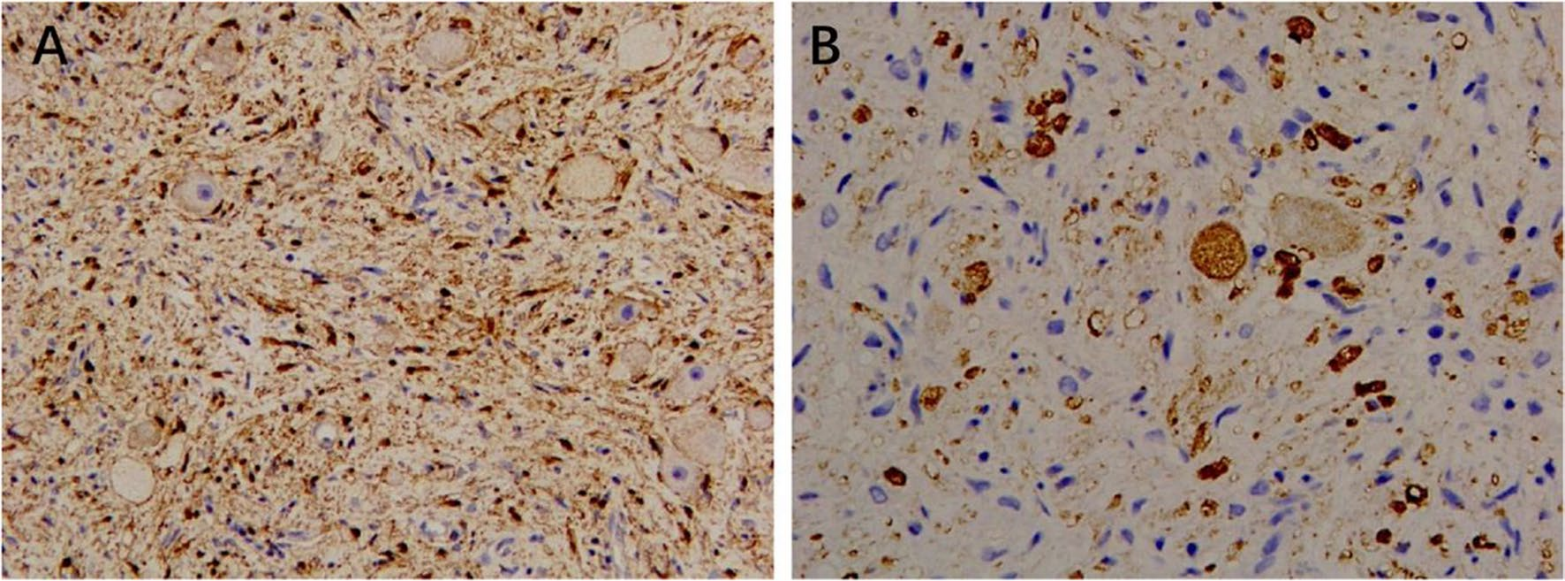
Immunohistochemical staining showed positive Schwann cell interstitial S-100 (**A**) and ganglion cells were NSE positive (**B**). Original magnification × 200

The pathological examination findings were as follows: A nodular mass, measuring 13 cm × 8 cm × 8 cm in size, with an intact capsule, was dissected; the cut surface was gray and yellow, solid, and soft in texture, without bleeding or necrosis (Fig. 2). The microscopic examination findings were as follows: The tumor was composed of nerve fibers and ganglion cells, and no neuroblastic components were observed. The ganglion cells were clustered or distributed among the nerve fibers (Fig. 3). The tumor area showed edema and degeneration along with ganglion cell degeneration, and the tumor envelope. Compressed adrenal tissue was observed outside the envelope. Immunohistochemical staining: Schwann cells were positive for S-100 (Fig. 4A), and ganglion cells were NSE-positive (Fig. 4B). Pathological diagnosis: ganglioneuroma.

No local recurrence was observed on ultrasound examination at the 1-year follow-up.

## Discussion

Ganglioneuroma is a benign tumor composed of relatively mature ganglion cells and nerve fibers and originates from neural crest cells [1]. Most GNs occur in patients aged > 10 years, and are most commonly observed in the posterior mediastinum, followed by the retroperitoneum [1]; a few cases also show GN occurring in the adrenal gland [1]. In a retrospective study from China, of the 15 patients with AGN, the mean size of the tumors as observed during the CT scan was 6.27 cm (range, 2.5–14 cm) [2]. In our case, the boy was presented with nausea and vomiting, and CT showed a large solid mass in the abdomen with a maximum diameter of the tumor was 13 cm.

In general, GNs have a clear perimeter and fibrous capsule, and the cut surface is grayish-white or grayish-yellow [1]. Histologically, GNs are categorized as mature and immature. The mature GNs are composed of mature Schwann cells and ganglion cells and completely lack neuroblasts, and the immature GNs include both immature and mature ganglion cells [3]. Immunophenotypically, ganglion cells express NSE and PGP9.5 and Schwann cells express S-100.

Recent studies have shown that all GNs express GATA3 [4], suggesting that GATA3 is a useful marker for GNs. Also, in GNs, the tyrosine kinase receptor *ERBB3* is one of the most commonly upregulated genes [5]. Compared to neuroblastomas, GNs do not show *MYCN* gene amplification [6].

The differential diagnostic criteria of GNs are as follows: (1) Ganglioneuroblastoma: It is categorized as nodular and mixed, both of which contain neuroblastic components. The nodular type contains neuroblastic components and ganglion cells, and there are clear boundaries between the neuroma components. In the mixed type, the neuroblastic components are irregularly distributed in the Schwann cell interstitium in the form of focal or small nests. The CT scan usually lacks specific features and the diagnosis depends on the pathological examination [1]. (2) Neurofibroma: The number of ganglion cells in the GNs is small. An abdominal CT scan usually shows a heterogeneous dense mass, ranging from about 30 to 50 HU [7]. A careful search can avoid misdiagnosis as a neurofibroma.

Biologically, GNs are benign tumors that rarely metastasize or recur. However, in a study of 49 cases, two cases showed metastasis to local lymph nodes and one case showed metastasis to soft tissue [6]. Occasionally, GNs can transform into malignant peripheral nerve sheath tumors [6]. Nevertheless, the prognosis after surgical resection of GN is good, and no postoperative adjuvant chemotherapy is required [8].

The surgical method can either be open or laparoscopic tumor resection. Laparoscopic surgery is an ideal surgical method for GNs because it is associated with lesser trauma and faster recovery [8, 9]. In our case, it was hard to differentiate the GN from neuroblastoma based on the CT image before the surgery. Removing the tumor was highly important for the diagnosis and deciding on further treatment (chemotherapy- or radiotherapy). Our patient underwent successful surgical treatment to remove the tumor and pathological examination showed that it was a benign tumor. The postoperative recovery was good. Since GN is benign in nature, it was not necessary to initiate the adjuvant therapy, including chemotherapy and radiotherapy.

Our case findings provide a new reference for the management of giant AGNs in children and recommend laparoscopic resection in patients with giant retroperitoneal tumors.

## Conclusion

Although AGN in children is a rare and benign disease, it should be considered when CT shows a solid mass in the retroperitoneal space with delayed enhancement. The ultimate diagnosis of GN depends on histopathological examination. The optimal treatment for GN is surgical excision.

## Data Availability

The original contributions presented in the study are included in the article/supplementary material, further inquiries can be directed to the corresponding author/s.

## Abbreviations

AGN: Adrenal ganglioneuroma
CT: Computed tomography
GN: Ganglioneuroma
